# Perspectives of the cohort of health professionals in the WiSDOM study on the learning environment, transformation, and social accountability at a South African University

**DOI:** 10.1080/10872981.2023.2185121

**Published:** 2023-03-07

**Authors:** Laetitia C. Rispel, Prudence Ditlopo, Janine White, Duane Blaauw

**Affiliations:** a Centre for Health Policy & South African Research Chairs Initiative (SARChI), School of Public Health, Faculty of Health Sciences, University of the Witwatersrand, Johannesburg, South Africa; b Centre for Health Policy, School of Public Health, Faculty of Health Sciences, University of the Witwatersrand, Johannesburg, South Africa; c School of Public Health, Faculty of Health Sciences, University of the Witwatersrand, Johannesburg, South Africa

**Keywords:** Learning environment, decolonisation, social accountability, transformation, health professions education, WiSDOM, South Africa

## Abstract

**Background:**

The dearth of empirical research on transformative health professions education informed this study to examine the factors that influence the perspectives of the cohort of health professionals in the WiSDOM study on the learning environment, transformation, and social accountability at a South African university.

**Methods:**

WiSDOM, a prospective longitudinal cohort study, consists of eight health professional groups: clinical associates, dentists, doctors, nurses, occupational therapists, oral hygienists, pharmacists, and physiotherapists. At study inception in 2017, participants completed a self-administered questionnaire that included four domains of selection criteria (6 items); the learning environment (5 items); redress and transformation (8 items); and social accountability (5 items). In the analysis, we, rescaled the original Likert scoring of 1 (strongly disagree) to 7 (strongly agree) to a new scale ranging from 0–10. We calculated the mean scores for each item and across items for the four domains, with low scores (0.00-1.99) classified as poor and high scores (8.00–10.00) as excellent. We used multiple linear regression analysis to compare the mean scores, while adjusting for different socio-demographiccharacteristics.

**Results:**

The mean age of the 501 eligible participants was 24.1 years; the majority female (72.9%), 45.3% self-identified as Black African; and 12.2% were born in a rural area. The domains of selection criteria and redress and transformation obtained mean scores of 5.4 and 5.3 out of 10 respectively, while social accountability and the learning environment obtained mean scores of 6.1 and 7.4 out of 10 respectively. Self-identified race influenced the overall mean scores of selection criteria, redress and transformation, and social accountability (*p* < 0.001). Rural birth influenced the perceptions on selection criteria, redress and transformation (*p* < 0.01).

**Conclusion:**

The results suggest the need to create inclusive learning environments that foreground redress, transformation, and social accountability, while advancing the discourse on decolonised health sciences education.

## Introduction

The 2021 World Health Assembly reaffirmed the objectives of the 2030 global strategy on human resources for health (HRH) to expand and transform the recruitment, development, education, training, distribution, retention and financing of HRH [1]. The curriculum and learning environment are central to the goal of transforming the education and training of health professionals, in order to strengthen health systems and to meet population health needs [2–4].

The learning environment refers to ‘the educational, physical, social, and psychological context in which trainees are immersed’ [5]. In the past five decades, the learning environment has remained an important focus of research, especially in medical schools [5]. In addition to its role in transforming health professional education [3], several studies have demonstrated the positive association between a supportive learning environment and student well-being, identity, values, academic success and professionalism [5–7]. In contrast, an unsupportive learning environment, especially in clinical settings, may lead to burnout, exhaustion, and cynicism [8,9]. This evidence led to the development of guidelines on a high-quality learning environment in the United States to combat the problems of burnout and depression and to ensure a strong medical education system able to contribute to optimal population health [10]. Importantly, the 2010 Lancet Global Commission on health professional education for the 21^st^ century posited that the learning environment and academic systems could influence health professionals’ future career decisions to specialise in tertiary health care facilities or to choose primary health care settings and service provision to under-served and/or disadvantaged communities [2].

There are numerous tools and approaches to assessing the learning environment. A 2014 systematic review that examined the validity of learning environment tools in medical schools found 15 unique ones [5]. Of these tools, the Dundee Ready Education Environment Measure (DREEM), with 50 items clustered into a five categories: perception of teachers; teaching; atmosphere; academic self-perception; and social self-perception [11], has had extensive utilisation as a learning environment diagnostic inventory across different geographical settings [5,12]. The DREEM has been criticised for validity inferiority [5] and its lack of granularity in identifying strategies to remedy identified problems [13]. Nonetheless, by 2014, DREEM accounted for 44% of all learning environment publications [5], and had been used in Australia [14], Spain [15], and several Asian [16–18] and African countries [19–21].

Several studies have demonstrated an association between socio-demographic variables and student perceptions of the different domains of the learning environment [7–9,15,22]. The role of context in student perceptions of the learning environment was illustrated in a large US multi-centre study that found that the medical school campus explained 15.6% of the total variance in the learning environment scores [23]. In African countries, studies found that resource availability played a key role in student perceptions of the learning environment [24,25].

Concomitant to the role of the learning environment in transformative health professional education is the discourse on social accountability and diversity [26]. Social accountability, refers to the obligation of health sciences education, research, and service activities to address the priority health needs of communities [27]. Other key aspects embedded in social accountability are graduate knowledge, skills and attitudes; inter-professional collaboration; partnerships with and immersion in communities; prioritisation of marginalised, vulnerable, and underserved communities; and population health improvements [28,29]. Several conceptual frameworks on social accountability, some with specific measures and indicators, have been developed to guide medical schools specifically [28–31] or health professional education institutions in general [32–34]. Although some have criticised the notion of social accountability as a conservative concept taken directly from business [35], its stated goal is transformative, namely to produce health professionals that are change agents in the health system [29].

Notwithstanding the dialectical relationship between the learning environment, social accountability and transformative health professional education [26], none of the commonly used learning environment tools focuses on social accountability [7,11,23]. However, there are nascent initiatives that suggest a more holistic and integrated approach to health professions education [4], while a new proposed model on social accountability includes principles of equity, diversity and inclusion [36].

The advancement of diversity of the health workforce is another vital aspect of transformative health professional education [26], combined with the imperative for structural reforms and redress to counter discrimination [37,38] and an explicit focus on decolonisation and decoloniality of health professions education, especially in low-and-middle income countries (LMICs) such as South Africa [39,40].

South Africa is a constitutional democracy, yet continues to suffer the legacy of its colonial and apartheid past of human rights violations, racial discrimination, and inequalities in access to resources and higher education [41]. The 2015 and 2016 *FeesMustFall* student protests at South African universities highlighted the ongoing struggle of financial access to higher education and its intersection with other aspects of transformation, notably race, gender, institutional culture and decolonisation of curricula and knowledge [39,42–44]. Similarly, social accountability has dominated the discourse on transformative medical education in South Africa [45,46] and has been extended to nursing education [47].

There are ongoing decolonisation debates in the health sciences [42], nursing [48], speech therapy and audiology [49], and psychology [50] in South Africa. However, there is a dearth of empirical studies that examine the perspectives of multiple groups of final year health professional students on different aspects of decolonised health sciences education. The opportunity for such an empirical investigation was presented by the WiSDOM (Wits longitudinal Study to Determine the Operation of the labour Market among its health professional graduates) study in South Africa. WiSDOM is a prospective longitudinal cohort study that aims to examine the long-term career choices (e.g., retention in the relevant profession) and job location decisions (e.g., urban vs rural practice) of health professional graduates of the University of the Witwatersrand (Wits) in Johannesburg, South Africa [51]. We have described elsewhere the detailed WiSDOM methodology [52] and baseline characteristics of cohort members [51].

The paper aims to describe the perspectives of the eight categories of health professionals in the WiSDOM cohort study on the learning environment, transformation, and social accountability, and the factors that influence these perspectives. The rationale for the paper is both its scholarly contribution, and its policy relevance. The paper contributes to and advances the discourse on a transformative learning environment, social accountability, and decolonisation of health sciences education in South Africa, as well as in other LMICs. The perspectives of health professionals in the cohort when they were final year students provide a baseline to compare the perspectives of other health professional students, suggest areas for improvement, and examine the association between these perspectives and long-term outcomes on career choices and job location decisions.

## Materials and methods

A brief overview of the WiSDOM cohort study methods is given below.

### Study design and population

**Figure 1.**
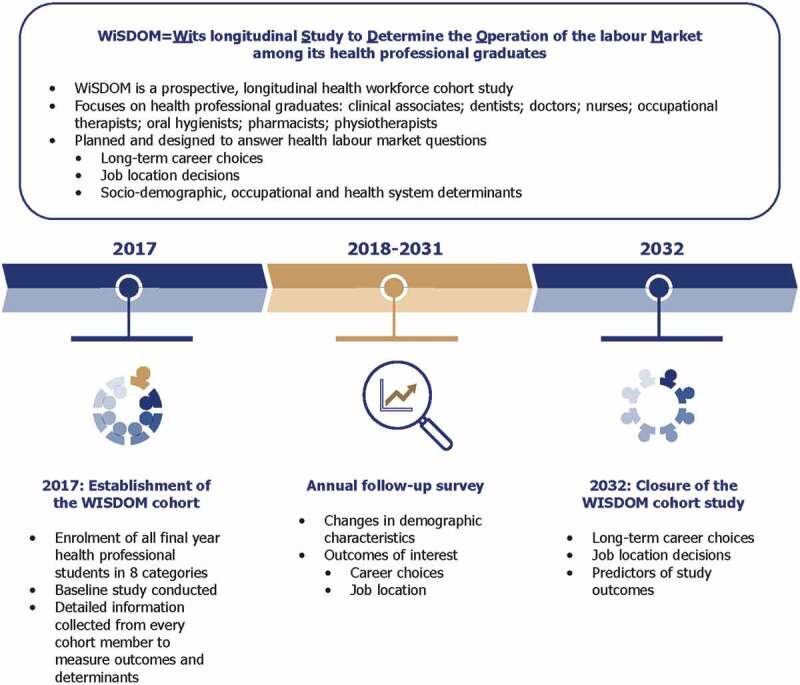
Overview of WiSDOM study design and research questions.

The WiSDOM study is a prospective, longitudinal cohort study that was designed to answer key health labour market questions on the long-term career choices and job location decisions of health professional graduates of Wits University in Johannesburg, South Africa. The cohort, established in 2017, consists of the eight health professional categories of clinical associates, dentists, doctors, nurses, occupational therapists, oral hygienists, pharmacists and physiotherapists trained in the Wits Faculty of Health Sciences [51]. The WiSDOM cohort will be followed up for 15 years. Figure 1 is a schematic overview of the research questions and design of the WiSDOM study.

### Data collection tool

Following an extensive literature review, the research team developed a self-administered questionnaire (SAQ) to meet the WiSDOM study objectives, specifically its main outcomes of tracking the long-term career choices and job location decisions of health professionals [51,52]. The original SAQ was divided into eight sections, summarised in Table 1.

**Table 1.** Sections of the self-administered questionnaire.

1 Detailed contact information: own, parents, spouse, friends 2 Demographic characteristics: age, gender, marital status, children, place of birth. We dealt with the contentious issue of race, by asking students to self-identify their race from a list of statistical categories prescribed by the South African government. 3 Financing of training 4 Perspectives on selection criteria for field of study, the learning environment, redress, reconciliation, transformation, and social accountability 5 Attitudes to internship, community, and rural service 6 Altruism and public service motivation 7 Career intentions-employment, profession, specialisation 8 Job location intentions – country, sector, geographical area, and facility type.

Below, we describe the development of and rationale for section 4 of the SAQ on a transformative learning environment, which is the focus of this paper.

Although the DREEM has been used extensively in different geographical settings, the five domains and 50 items appear to be ahistorical [11], with no reference to the context of learning, or transformation and/or decolonisation discourses. Similarly, the Johns Hopkins Learning Environment Scale with seven factors/subscales and 28 items excludes any focus on context, transformation, and/or social accountability [7].

The research team considered it important to focus on student perspectives of transformation and social accountability as a critical part of the learning environment, for two main reasons. Firstly, the study commenced in 2017, two years after the first *#FeesMustFall* protests [53]. These protests began in mid-October 2015 in South Africa and continued into 2016. Led by students, the goals of protests were to stop increases in student fees, increase government funding of universities, and advance the student campaign for the decolonisation of higher education [53,54]. This means that the WiSDOM cohort members had lived experiences of the 2015 and 2016 *FeesMustFall* protests. Secondly, the protests were an important part of the context of health professions education. Hence, we wanted the SAQ to capture these experiences and the context.

Consequently, the research team developed a tool with four domains and items to take account of the context of health sciences education in South Africa in 2017, namely the student protests in the preceding two years, the ongoing discourse on transformation and the role of students as epistemological agents. These four domains are shown in Table 2 and consist of the selection criteria for the relevant field of study (6 items); the learning environment (5 items); redress, and transformation (8 items); and social accountability (5 items).

**Table 2.** Overview of domains and items on transformative learning environment.

**Selection criteria (6 items)** I believe the selection criteria are fair I think the selection criteria favour white applicants I think the selection process is too subjective I think the selection criteria discriminates against older applicants I think the selection criteria are not transparent I think the selection criteria do not sufficiently address historical disparities
**Learning environment (5 items)** I think Wits offers the best curriculum in my field of study My curriculum encourages critical thinking I think Wits has high quality lecturers I think Wits has good teaching facilities I believe that Wits training gives its graduates the edge in getting employment
**Redress and transformation (8 items)** My curriculum is too European The Faculty of Health Sciences is sensitive to the needs of black students I feel that the institutional culture in the Faculty does not promote racial diversity The Faculty is not doing enough to ensure that the lecturer profile is representative of the demographics of the country I think black students are made to feel less competent than white students There is open support of people regardless of their sexual orientation The Faculty does not take gender equality seriously I think Wits does not do enough for students who are struggling financially
**Social accountability (5 items)** I think the Wits curriculum equips me to work in resource-poor environment My curriculum is biased towards specialisation My curriculum does not foster teamwork My curriculum encourages accountability to the community I feel that Wits training prepares its graduates for the reality of working in the South African public service

Each of these items was measured on a Likert scale as follows: 1 (strongly disagree), 2 (disagree), 3 (slightly disagree), 4 (neither agree nor disagree), 5 (slightly agree), 6 (agree), 7 (strongly agree).

### Validity and reliability

Prior to data collection, we established content and face validity of the SAQ. We convened a consultative workshop with stakeholders from each of the eight professional groups to present the draft SAQ, and to elicit their inputs [52]. We also piloted the SAQ with nursing, medical and clinical associate students in their penultimate study year. Both the stakeholders and pilot students expressed support for WiSDOM and suggested minor rephrasing of the questions.

We computed Cronbach’s Alpha coefficients to determine the reliability and coherence [55] between the items developed to measure the student perspectives on the learning environment, transformation and social accountability. These coefficients were 0.70 or higher demonstrating internal consistency and reliability of the domains and items: Field of Study (FOS) selection criteria (α = 0.738); learning environment (α = 0.728); redress and transformation (α = 0.852); and social accountability (α = 0.726).

### Data collection

We conducted the baseline survey between July and September 2017, with separate data collection sessions for each of the eight professional groups that constitute the WiSDOM cohort [51,52]. The details of data collection are described in two previous publications [51,52], but are summarised here.

We obtained voluntary, informed consent from each participant. The data collection occurred during an appropriate timeslot in the final year academic programme in a computer laboratory or e-learning room at the Wits Health Sciences Campus located in Johannesburg, South Africa [51,52]. Each study participant completed the SAQ using REDCap (Research Electronic Data Capture), an online database system, designed to collect, store, secure, organise, and analyse data [56].

### Data analysis

The data were imported from REDCap into Stata® 16 for analysis. To simplify interpretation of the 7-level Likert scale responses we undertook two different data transformations. Firstly, we combined the ‘slightly agree’, ‘agree’ and ‘strongly agree’ responses to compute the proportion of respondents who agreed with each statement. Secondly, we reversed the coding of all negatively phrased items, and rescaled the original Likert scoring of 1–7 to a new scale ranging from 0–10 using the formula:

$$Score_{New}=\frac{\left(Score_{Likert}-1\right)}{6}\times 10$$

The mean scores were then calculated for each single item and across the items for each of the four domains. We also used principal component analysis (PCA) to calculate a weighted index for each domain, but these indices were highly correlated with the equally weighted means. Hence, the results reported here use the standard means.

We classified the overall mean scores out of 10 for each domain as follows: 0.00–1.99 poor; 2.00–3.99 below average; 4.00–5.99 average; 6.00–7.99 good; and 8.00–10.00 excellent.

The results are presented separately for each professional field of study (FOS) and for the entire cohort. Although the cohort mean is influenced by the unequal distribution among professional groups, it represents the opinions of health professional students graduating from Wits University in 2017.

We used multiple linear regression analysis to compare the mean scores for each domain between the professional groups, while adjusting for different socio-demographic characteristics between groups.

## Results

### Demographic characteristics

At baseline in 2017, we recruited 511 final year health professional students, and obtained an 89.5% response rate [51]. Although we have described the demographic characteristics of the entire cohort in the baseline paper [51], the cohort included ten medical students who studied in Cuba, and only joined the Wits medical programme in the final 18 months. As these ten students were unable to comment on the learning environment, transformation, and social accountability, they are excluded from the results presented. Hence, the socio-demographic characteristics of the eligible participants for this paper are shown in Table 3.

**Table 3.** Socio-demographic characteristic of participants.

		CA	DT	MD	NS	OT	OH	PH	PT	Total
		(n=44)	(n=17)	(n=272)	(n=21)	(n=36)	(n=7)	(n=58)	(n=46)	(n=501)
Age	(Mean ± SD)	21.9 ± 1.7	25.4 ± 3.5	25.2 ± 2.1	23.0 ± 1.1	22.6 ± 0.9	21.3 ± 0.9	22.7 ± 1.7	22.9 ± 1.3	24.1 ± 2.3
Female	(%)	86.4%	76.5%	65.8%	81.0%	100.0%	100.0%	62.1%	84.8%	72.9%
Self-identified	Black African	93.0%	11.8%	40.6%	81.0%	6.1%	71.4%	68.4%	18.2%	45.3%
Race (%)	Coloured	0.0%	5.9%	2.6%	0.0%	0.0%	0.0%	1.8%	13.6%	3.1%
	Asian/Indian	2.3%	64.7%	17.7%	0.0%	21.2%	14.3%	26.3%	15.9%	18.2%
	White	4.7%	17.6%	39.1%	19.0%	72.7%	14.3%	3.5%	52.3%	33.4%
Born rural	(%)	13.6%	0.0%	10.7%	19.0%	2.8%	14.3%	29.3%	6.5%	12.2%
Previous degree	(%)	6.8%	35.3%	22.1%	0.0%	5.6%	0.0%	5.2%	10.9%	15.8%

Legend: CA=clinical associate; DT=dentist; MD=medical doctor; NS=nurse; OH=oral hygienist; OT=occupational therapist; PH=pharmacist; PT=physiotherapist.

The mean age of the 501 eligible participants was 24.1 years; the majority female (72.9%), 45.3% self-identified as Black African; 12.2% were born in a rural area, and 15.8% had a previous degree.

### Student perspectives on the learning environment, transformation, and social accountability

We asked the study participants whether they were familiar with the selection criteria for the relevant FOS or health profession. Only 42.71% (*n* = 214) of the eligible cohort members agreed that they were. Hence, the responses for the six selection criteria items are reported for this group only.

The remainder of the items did not require familiarity with the selection criteria; hence the data reflect the perspectives of the cohort members who completed the questions. Table 4 shows a wide range of perspectives across the eight professional groups for each of the four domains.

**Table 4.** Student perceptions of learning environment, transformation and social accountability.

	% Agreeing								
	CA	DT	MD	NS	OT	OH	PH	PT	Total
**Selection Criteria**	**(*n* = 24)**	**(*n* = 5)**	**(*n* = 91)**	**(*n* = 16)**	**(*n* = 24)**	**(*n* = 4)**	**(*n* = 28)**	**(*n* = 22)**	**(*n* = 214)**
I believe the selection criteria are fair	79.2%	20.0%	36.3%	87.5%	62.5%	75.0%	42.9%	54.6%	50.9%
I think the selection criteria favour white applicants	0.0%	0.0%	33.0%	0.0%	20.8%	0.0%	25.0%	27.3%	22.4%
I think the selection process is too subjective	4.2%	100.0%	46.2%	18.8%	33.3%	75.0%	39.3%	40.9%	38.3%
I think the selection criteria discriminates against older applicants	4.2%	0.0%	24.2%	25.0%	37.5%	25.0%	17.9%	9.1%	20.6%
I think the selection criteria are not transparent	20.8%	80.0%	78.0%	25.0%	41.7%	50.0%	64.3%	45.5%	57.9%
I think the selection criteria do not sufficiently address historical disparities	29.2%	20.0%	47.3%	25.0%	37.5%	50.0%	28.6%	27.3%	37.4%
**Learning environment**	**(*n* = 44)**	**(*n* = 17)**	**(*n* = 272)**	**(*n* = 21)**	**(*n* = 36)**	**(*n* = 7)**	**(*n* = 58)**	**(*n* = 46)**	**(*n* = 501)**
I think Wits offers the best curriculum in my field of study	38.6%	64.7%	68.0%	95.2%	75.0%	71.4%	41.4%	95.7%	66.5%
My curriculum encourages critical thinking	79.6%	82.4%	80.5%	95.2%	94.4%	57.1%	70.7%	100.0%	82.4%
I think Wits has high quality lecturers	79.6%	88.2%	93.8%	100.0%	97.2%	42.9%	69.0%	100.0%	89.8%
I think Wits has good teaching facilities	95.5%	52.9%	92.7%	95.2%	97.2%	100.0%	86.2%	97.8%	91.8%
I believe that Wits training gives its graduates the edge in getting employment	79.6%	47.1%	70.6%	95.2%	77.8%	57.1%	41.4%	91.3%	70.5%
**Redress and Transformation**	**(*n* = 44)**	**(*n* = 17)**	**(*n* = 272)**	**(*n* = 21)**	**(*n* = 36)**	**(*n* = 7)**	**(*n* = 58)**	**(*n* = 46)**	**(*n* = 501)**
My curriculum is too European	13.6%	11.8%	26.1%	23.8%	8.3%	28.6%	19.0%	6.5%	20.6%
The Faculty of Health Sciences is sensitive to the needs of black students	22.7%	47.1%	34.9%	23.8%	61.1%	42.9%	31.0%	60.9%	37.7%
I feel that the institutional culture in the Faculty does not promote racial diversity	52.3%	23.5%	42.7%	52.4%	36.1%	57.1%	50.0%	34.8%	43.1%
The Faculty is not doing enough to ensure that the lecturer profile is representative of the demographics of the country	56.8%	17.7%	38.2%	23.8%	22.2%	71.4%	56.9%	10.9%	37.5%
I think black students are made to feel less competent than white students	38.6%	11.8%	48.2%	57.1%	22.2%	57.1%	36.2%	21.7%	40.9%
There is open support of people regardless of their sexual orientation	72.7%	64.7%	46.7%	71.4%	80.6%	42.9%	62.1%	84.8%	58.3%
The Faculty does not take gender equality seriously	15.9%	23.5%	29.8%	4.8%	13.9%	28.6%	17.2%	6.5%	22.6%
I think Wits does not do enough for students who are struggling financially	77.3%	70.6%	67.3%	57.1%	52.8%	85.7%	72.4%	65.2%	67.5%
**Social accountability**	**(*n* = 44)**	**(*n* = 17)**	**(*n* = 272)**	**(*n* = 21)**	**(*n* = 36)**	**(*n* = 7)**	**(*n* = 58)**	**(*n* = 46)**	**(*n* = 501)**
I think the Wits curriculum equips me to work in resource-poor environment	63.6%	52.9%	57.7%	71.4%	88.9%	85.7%	55.2%	100.0%	64.9%
My curriculum is biased towards specialisation	31.8%	47.1%	68.8%	19.1%	33.3%	28.6%	31.0%	19.6%	50.7%
My curriculum does not foster teamwork	4.6%	29.4%	26.5%	4.8%	5.6%	0.0%	6.9%	2.2%	17.4%
My curriculum encourages accountability to the community	81.8%	70.6%	41.2%	85.7%	77.8%	71.4%	70.7%	95.7%	59.1%
I feel that Wits training prepares its graduates for the reality of working in in the South African public service	86.4%	58.8%	71.3%	95.2%	83.3%	71.4%	62.1%	93.5%	75.1%

CA=clinical associate; DT=dentist; MD=medical doctor; NS=nurse; OH=oral hygienist; OT=occupational therapist; PH=pharmacist; PT=physiotherapist.

On the domain of selection criteria, 50.9% of the entire cohort perceived the selection criteria for their FOS to be fair, with the lowest perception among dentists at 20.0%, compared to 87.5% of nurses. Overall, 38.3% of the cohort thought the selection process is too subjective, ranging from 4.2% of clinical associates to 100.0% of dentists. On the issue of transparency, 57.9% of all cohort members believed the selection criteria are not transparent, ranging from 20.8% of clinical associates to 78.0% of doctors and 80.0% of dentists. On whether the selection criteria favour whites, 22.4% of the cohort thought so, with variation across the eight professional groups. Similarly, 37.4% of cohort members perceived the selection criteria insufficient in addressing historical disparities, ranging from 20.0% of dentists to 50.0% of oral hygienists.

The perspectives of the WiSDOM’s cohort on the domain of the learning environment also revealed wide-ranging perspectives.

The majority of cohort members (66.5%), believed that the Wits curriculum is the best, but this ranged from of 38.6% clinical associates to 95.7% of physiotherapists. Similarly, 82.4% of the WiSDOM cohort thought that the curriculum encourages critical thinking, but oral hygienists were the least enthusiastic (57.1%). The course lecturers (89.8% agreement) and teaching facilities (91.8%) obtained high levels of agreement, with some differences by health professional categories (Table 4).

Wits University has a slogan that states ‘Wits gives graduates the edge’, 70.5% of cohort members agreed with the statement regarding employment, ranging from 41.4% of pharmacists to 95.2% of nurses.

Regarding the domain of redress and transformation, 20.6% of cohort members agreed that the relevant curriculum is too Eurocentric, ranging from 6.5% of physiotherapists to 28.6% of oral hygienists. However, 37.7% of all participants believed that the Wits Faculty of Health Sciences (FHS) is sensitive to the needs of black students, and 40.9% agreed with a statement that black students are made to feel less competent than white students. Although 43.1% of cohort members agreed with a negative statement that the FHS does not promote racial diversity, clinical associates (52.3%), nurses (52.4%), oral hygienists (57.1%) and pharmacists (50.0%) were more negative about the promotion of racial diversity in the FHS, compared to dentists (23.5%), occupational therapists (36.1%), physiotherapists (34.8%) or doctors (42.7%). Overall, 37.5% agreed also with a negatively phrased statement that the FHS is not transforming the demographic profile of lecturers.

The majority of cohort members (58.3%) agreed with a statement that there is open support of people regardless of sexual orientation. Only 22.6% of cohort members agreed with the negatively phrased question that the FHS does not take gender equality seriously, ranging from 4.8% of nurses to 29.8% of doctors.

The majority (67.5%) of all cohort members agreed with a statement that the university does not do enough for financially struggling students, ranging from 52.8% of occupational therapists to 85.7% of oral hygienists.

The cohort members also expressed a range of views on the domain of social accountability. The majority of cohort members (64.9%) agreed with a statement that the relevant curriculum prepares graduates for working in resource-poor environment, ranging from 52.9% of dentists to 100.0% of physiotherapists. Overall, 50.7% agreed with a statement that the curriculum is biased towards specialisation (19.6% of physiotherapists, 68.8% of doctors). On the negatively phrased question that the curriculum does not foster teamwork, only 17.4% of cohort members agreed, ranging from 0.0% of oral hygienists, to 29.4% of dentists. The majority of cohort members (59.1%) agreed with a statement that the curriculum, encourages accountability to communities, ranging from 41.2% of doctors to 95.7% of physiotherapists. On whether the university prepares graduates for the reality of work in the public sector, overall, 75.1% of cohort members agreed with this statement, ranging from 58.8% of dentists to 95.2% of nurses.

### Summary scores for the four domains

**Figure 2.**
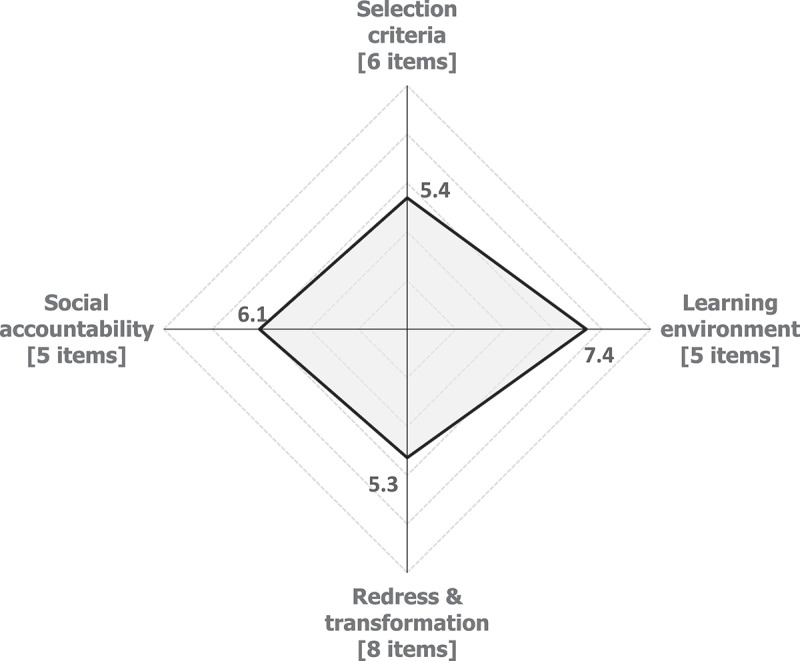
Spider plots showing overall mean scores by domain.

Figure 2 shows the mean scores out of 10 for each domain for the entire cohort. Overall, the domains of selection criteria and redress and transformation obtained mean scores of 5.4 and 5.3 out of 10 respectively, which we categorised as average. The domain of social accountability obtained a good score of 6.1 out of 10, while the learning environment obtained the highest score of 7.4 out of 10.

CA=clinical associate; DT=dentist; MD=medical doctor; NS=nurse; OH=oral hygienist; OT=occupational therapist; PH=pharmacist; PT=physiotherapist

**Figure 3.**
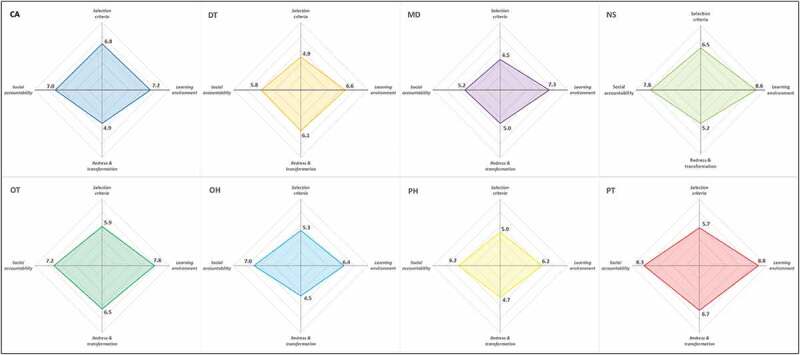
Spider plots showing overall mean scores by domain and health professional category.

Figure 3 shows the mean scores out of 10 for each domain by health professional category.

CA=clinical associate; DT=dentist; MD=medical doctor; NS=nurse; OH=oral hygienist; OT=occupational therapist; PH=pharmacist; PT=physiotherapist

Figure 3 highlights the differences in perceptions across the eight health professions. Only the clinical associate (6.84 ± 1.52) and nursing (6.55 ± 1.51) programmes obtained good overall mean scores for the domain of selection criteria, with average scores for all other programmes. The nursing (8.62 ± 1.01) and physiotherapy (8.76 ± 0.79) programmes obtained excellent overall mean scores for the learning environment domain, but lower scores by dentists (6.59 ± 1.74), oral hygienists (6.43 ± 1.99), and pharmacists (6.22 ± 1.71).

The domain of redress and transformation obtained average scores for the majority of programmes, with the exception of dentistry (6.05 ± 1.74), occupational therapy (6.49 ± 1.67) and physiotherapy (6.68 ± 1.60).

The social accountability domain obtained good or excellent overall mean scores, except for dentistry (5.84 ± 2.09) and medicine (5.25 ± 1.76) that were average.

### Factors influencing the WiSDOM cohort perspectives

Table 5 shows the factors influencing the cohort perspectives on selection criteria, the learning environment, redress and transformation and social accountability in the multiple linear regression. As can be seen health professional category or field of study was statistically associated with the perspectives of cohort members on the four domains. For example, the medical group was significantly less positive than other groups about selection as well as redress and transformation, physiotherapists rated the learning environment lower, and occupational therapists scored higher on three of the four domains.

**Table 5.** Factors that influence WiSDOM cohort perceptions.

		1	2	3	4
Predictor		Selection score	Learning environment score	Redress & transformation score	Social Accountability score
**Field of** **study** (compared to unweighted mean of all FOS)	CA	1.506 ***	−0.406	−0.336	−0.560
		[<0.001]	[0.069]	[0.281]	[0.146]
	DT	−1.062	−0.476	0.326	0.637
		[0.116]	[0.143]	[0.472]	[0.256]
	MD	−1.058 ***	0.209	−0.630 **	−0.515
		[0.001]	[0.177]	[0.004]	[0.054]
	NS	1.104 **	1.206 ***	−0.315	−0.468
		[0.006]	[<0.001]	[0.431]	[0.344]
	OT	0.096	0.462 *	0.908 **	1.133 **
		[0.787]	[0.050]	[0.006]	[0.005]
	OH	−0.176	−1.225 *	−0.543	−0.904
		[0.817]	[0.011]	[0.418]	[0.276]
	PH	−0.334	−1.193 ***	−0.520	−0.726*
		[0.313]	[<0.001]	[0.059]	[0.034]
	PT	−0.076	1.424 ***	1.109 ***	1.403 ***
		[0.828]	[0.000]	[0.000]	[0.000]
**Age**		−0.100	−0.098 *	−0.008	−0.076
		[0.303]	[0.033]	[0.877]	[0.149]
**Female**		0.217	0.158	−0.173	−0.053
		[0.437]	[0.295]	[0.323]	[0.760]
**SES index**		−0.178	−0.108	−0.179 **	−0.169 **
		[0.088]	[0.055]	[0.006]	[0.009]
**Born rural**		−1.314 **	−0.111	−0.447	−0.102
		[0.008]	[0.679]	[0.151]	[0.739]
**Previous degree**		0.350	0.042	0.134	0.428
		[0.438]	[0.862]	[0.632]	[0.122]
**Race**	Black African	—	—	—	—
	Coloured	−0.429	0.515	1.729 ***	0.880 *
		[0.604]	[0.187]	[<0.001]	[0.049]
	Asian/Indian	0.635	−0.008	2.368 ***	1.011 ***
		[0.076]	[0.967]	[<0.001]	[0.000]
	White	1.479 ***	−0.274	3.249 ***	0.973 ***
		[<0.001]	[0.114]	[<0.001]	[<0.001]
**Constant**		8.629 ***	9.117 ***	4.871 ***	8.388 ***
		[0.000]	[0.000]	[0.000]	[0.000]
***Observations***		*206*	*485*	*485*	*485*
***R-squared***		*0.340*	*0.216*	*0.449*	*0.345*
***p value***		*<0.001*	*<0.001*	*<0.001*	*<0.001*

Coefficient with p value in brackets.

*** *p*<0.001, ** *p*<0.01, * *p*<0.05. Legend: CA=clinical associate; DT=dentist; MD=medical doctor; NS=nurse; OH=oral hygienist; OT=occupational therapist; PH=pharmacist; PT=physiotherapist.

The scores for all domains decreased with advancing age, but this was only statistically significant for the learning environment measure. There was no statistically significant association between gender or having completed a previous degree and the domain scores. Higher socio-economic status was associated with lower scores for transformation and social accountability, while those born in rural areas scored significantly lower for the selection domain. Those individuals who self-identified as Coloureds, Indians and Whites scored significantly higher than self-identified Black African students in the scores for the transformation and social accountability domains. White students were also significantly more positive in their rating of selection.

## Discussion

In this paper, we have described the perspectives of the WiSDOM health professional cohort at Wits University in South Africa on the domains of selection criteria, learning environment, redress and transformation, and social accountability and the factors that influence these perspectives. These perspectives are likely to differ from those of subsequent cohorts of health professional graduates, and this is a limitation. However, WiSDOM remains novel as it is one of the few health workforce cohort studies in a low-and middle-income setting in Africa. We obtained a high response rate of 89.5% at baseline in 2017 [52]. The WiSDOM study enables us to examine how these perspectives influence long-term career choices and job location decisions, which are the main outcomes of interest.

Importantly, in this paper we advance the discourse on transformative health sciences education in several ways. First, we bring together the perspectives of health professional students on these four domains, rather than present perspectives only on the learning environment. Second, we developed a context-specific tool, which measures student perspectives on diversity regarding gender, sexual orientation, and race, as well as on social accountability. The tool has global relevance and could be adapted by researchers in other geographical settings. Third, we compare the perspectives of eight health professional categories, rather than maintain the dominant focus on medical students. This is important as transformative health professional education emphasises teamwork and multi-disciplinarity [2].

Although the selection of health professional students plays an important role in the equitable deployment and distribution of the health workforce [26], the commonly used learning environment tools do not focus on student perceptions of selection criteria [7,11]. Hence, our study is one of the first to examine student perceptions of the actual selection criteria for their FOS. We found that only 42.71% of the eligible cohort members were familiar with the selection criteria. Notwithstanding differences across the eight groups, our findings suggest that among this minority of students, there were widespread perceptions of unfairness, subjectivity, lack of transparency, and insufficient efforts by the Wits FHS to address the historical disparities, created by the brutal apartheid system.

However, Wits University has introduced numerous changes in the admission and selection criteria for health professional students to reflect diversity and to achieve equity [57]. In the multiple regression, perceptions of the selection criteria were associated with certain fields of study (clinical associates and nurses were more positive on average while medical students were more negative), students born in a rural area scored lower, and self-identified White ‘race’ scored significantly higher than Black Africans. These negative perceptions about selection could be related to the lack of communication and/or they could indicate the intense competition for entry into the undergraduate medical programme at Wits University. These findings have implications for the Wits FHS to make selection criteria more explicit and to raise awareness of the rationale for each FOS selection criteria.

Although the domain of the learning environment obtained a good overall mean score, our findings highlight the variations across the eight health professions, and the areas that require attention. The clinical associate and oral hygiene curricula obtained the lowest scores (Figure 3). A possible explanation of these negative perceptions of students is that the clinical associate FOS is a relatively new and evolving programme that commenced in 2009 [58]. Nonetheless, these issues reflected in the low scores need to be addressed by the FHS. Although Wits University is one of the few universities that offer the degree in oral hygiene, a small number of oral hygienists is trained, the division is small and part of the dental school, and there are limited posts available in the public health sector [59]. Dental students were particularly dissatisfied with their teaching facilities (Table 4), suggesting the need for improvements.

Age was the only variable that influenced perceptions of the learning environment, with older individuals giving lower scores. These differences could be related to maturity levels, varied life experiences and unmet expectations, resulting in more critical views. Although not comparable, another South African study found that age did not influence perceptions of the DREEM scores, but younger medical students ranked general surgery higher compared to older students [19]. In contrast, an Australian study among students of eight health science courses found that those students who enrolled in their course directly after completing high school provided less positive ratings on some DREEM subscales than students who enrolled for their course at a later stage [14]. Further qualitative research with final year health professional students should be done to explore why age influences student perceptions of the learning environment.

The perspectives of the WiSDOM cohort on the domain of redress and transformation underscore both the paradox and ongoing contestations regarding redress and transformation since South Africa’s democratic transition in 1994 [60]. Overall, one in five WiSDOM cohort members (20.6%) felt that the relevant curriculum is too Eurocentric (Table 4). Our findings provide empirical evidence to support the recommendations of the Academy of Science of South Africa [46] and the 2030 Human Resources for Health Strategy of South Africa [61] for the transformation of health professional curricula that meet the needs of communities and the health system in South Africa. Paton and colleagues (2020) have proposed an intersectional frame to disrupt existing models of health professions education and practice [62]. They have underscored the role of both institutions and individual faculty members in driving deloniality, transformation, and social justice [62]. At institutional level proposed strategies include dedicated resources, continuing professional development, and ensuring accountability, while calling faculty members to engage in critical and self-reflexivity, questioning assumptions and unlearning certain behaviours [62].

The perspectives of the WiSDOM cohort on the domain of redress and transformation also reflect the progress that has been made regarding diversity, namely gender, sexual orientation, and promotion of racial diversity. At the same time, the perspectives reflect the alienation that many Black African students continue to experience, namely lack of sensitivity to their needs, a feeling of being less competent than their white counterparts, and ongoing financial struggles. In the regression the redress and transformation scores for Black Africans were significantly lower than the other groups (Table 5). Our study provides empirical evidence of the degree of alienation felt by Black African students surveyed in 2017, which has been highlighted by other scholars [63,64].

A large proportion of cohort members (67.5%) indicated that the university’s support for students who struggle financially is inadequate. These perspectives were influenced by FOS, socio-economic status, and self-identified race. This could be related to the FeesMustFall protests in the two years preceding the baseline study, as financial access or exclusion ignited the protests [53]. Furthermore, as reported in the WiSDOM article on cohort baseline characteristics, a sizeable proportion of oral hygienists (57.1%), clinical associates (29.5%), nurses (29.6%) and pharmacists (39.7%) were dependent on some form of loan to complete their studies [51]. We acknowledge that FeesMustFall protests signalled the unfinished agenda of transforming the higher education system, which requires a national solution and government leadership. However, the university plays an important role in mediating financial access for students, hence creative strategies should be explored.

The domain of social accountability obtained a good overall mean score from the WiSDOM cohort. The cohort perspectives were influenced by FOS, socio-economic status, and self-identified race. The lowest overall mean score was obtained for the medical programme (5.25 ± 1.76), which has remained the focus of robust debates on the reforms needed to enhance the social accountability of medical graduates to meet the needs of the most vulnerable and marginalised communities, especially those in rural areas [45,46].

The student perspectives on all four domains were influenced to some extent by self-identified race, which remains an intensely contested issue in South Africa [60]. However, the country’s apartheid legacy and racism continue to shape socio-economic status, and access to social services, and universities remain a microcosm of society [63]. Hence, more efforts are needed in the Wits FHS to have ongoing discussions as well as practical initiatives to overcome the vestiges of structural and institutional racism. Adonis and Silinda (2021) have argued for transformation of institutional cultures because these represent ‘the effects of the values, attitudes, styles of interaction, and collective memories of a university’ [63]. We recommend that the voices of students should be central to that process of transformation, by acknowledging their experiences of marginalisation and alienation. The FHS has a moral obligation to create an environment of inclusion and sense of belonging, while building on its progress and achievements in the post-apartheid period.

## Conclusion

The 2022 report of the WHO director-general to the World Health Assembly underscores the criticality of the health workforce, and the contribution of their education to health systems transformation [65].

In this study, we foreground and present the perspectives of eight categories of health professional students, thus placing them at the centre of the discourse on these issues. The methodological strength of the WiSDOM cohort study enables the generation of empirical evidence on the complex issues of selection criteria, the learning environment, redress and transformation, and social accountability. Furthermore, the WiSDOM study allows for comparisons and the development of new hypothesis on health professions education. Over time, the WiSDOM study will enable us to examine the associations between the cohort perspectives on redress, transformation, and social accountability at baseline, with the long-term career choices and job location decisions of these health professionals. Ultimately, the future findings of the WiSDOM study can and should shape the transformation agenda in health sciences education and inclusive learning environments.

## Data Availability

Data cannot be shared publicly because this is an active cohort study of health professional graduates who are being followed up every year. The Human Research Ethics Committee (Medical) of the University of the Witwatersrand has imposed restrictions because of the confidential and sensitive nature of the data. Email: HREC-Medical.ResearchOffice@wits.ac.za; Site: https://www.wits.ac.za/research/researcher-support/research-ethics/ethics-committees/
